# Cecal microbiota composition differs under normal and high ambient temperatures in genetically distinct chicken lines

**DOI:** 10.1038/s41598-023-43123-9

**Published:** 2023-09-25

**Authors:** Philip M. Campos, Lori L. Schreier, Monika Proszkowiec-Weglarz, Sami Dridi

**Affiliations:** 1 USDA-ARS Research Participation Program, Oak Ridge Institute for Science and Education (ORISE), 1299 Bethel Valley Rd, Oak Ridge, TN 37830 USA; 2 USDA-ARS, NEA, Beltsville Agricultural Research Center, Animal Biosciences and Biotechnology Laboratory, 10300 Baltimore Avenue, Bldg. 307, BARC-East, Beltsville, MD 20705 USA; 3 USDA-ARS, NEA Bioinformatics, Statistics Group, 10300 Baltimore Ave, Bldg. 003, Rooms 229E, 330, 331; BARC-West, Beltsville, MD 20705 USA; 4 USDA-ARS, NEA, Beltsville Agricultural Research Center, Animal Biosciences and Biotechnology Laboratory, 10300 Baltimore Avenue, Bldg. 307, Room 304, BARC-East, Beltsville, MD 20705 USA; 5 USDA-ARS, NEA, Beltsville Agricultural Research Center, Animal Biosciences and Biotechnology Laboratory, 10300 Baltimore Avenue, Bldg. 307, Room 335, BARC-East, Beltsville, MD 20705 USA; 6 Center of Excellence for Poultry Science, University of Arkansas, 1260 W. Maple Street, Fayetteville, AR 72701 USA

**Keywords:** Microbiome, Agricultural genetics

## Abstract

Modern broilers, selected for high growth rate, are more susceptible to heat stress (HS) as compared to their ancestral jungle fowl (JF). HS affects epithelia barrier integrity, which is associated with gut microbiota. The aim of this study was to determine the effect of HS on the cecal luminal (CeL) and cecal mucosal (CeM) microbiota in JF and three broiler populations: Athens Canadian Random Bred (ACRB), 1995 Random Bred (L1995), and Modern Random Bred (L2015). Broiler chicks were subjected to thermoneutral TN (24 °C) or chronic cyclic HS (8 h/day, 36 °C) condition from day 29 until day 56. HS affected richness in CeL microbiota in a line-dependent manner, decreasing richness in slow-growing JF and ACRB lines, while increasing richness in faster-growing L1995 and L2015. Microbiota were distinct between HS and TN conditions in CeL microbiota of all four lines and in CeM microbiota of L2015. Certain bacterial genera were also affected in a line-dependent manner, with HS tending to increase relative abundance in CeL microbiota of slow-growing lines, while decreases were common in fast-growing lines. Predictive functional analysis suggested a greater impact of HS on metabolic pathways in L2015 compared to other lines.

## Introduction

Broiler chickens have been selected for high growth rate and feed efficiency over the past 80 years, leading to remarkable progress in breast yield and reduction of market age^1,2^. However, selection is not without trade-offs, where progress in growth and feed efficiency traits may come at the expense of other biological traits, such as immunity and gut integrity^2,3^. As a result, modern broilers are prone to environmental or bacterial challenges, leading to more significant negative effects under challenging conditions and negating the positive effects from improved performance traits.

One environmental challenge with a strong adverse effect on broilers is heat stress (HS). The negative consequences of HS are especially influential in avian species, due to physiological traits such as feathers and higher body temperature^4^. Core body temperatures are increased in broilers during HS, reducing performance and harming gastrointestinal tract (GIT) integrity, which can provoke immune responses^5^. Moreover, HS can affect the GIT microbiota by altering the bacterial composition and diversity^6,7^, which may lead to dysbiosis or leaky gut syndrome.

Bacterial communities within the GIT microbiota have been found to play a role in broiler health, affecting factors such as nutrient exchange, immune system modulation, digestive system physiology, feed efficiency, and pathogen exclusion^8–10^. Thus, we hypothesized that alterations to the GIT microbiota under HS may alter bacterial composition and the metabolic functioning of the microbiota, leading to declines in growth performance and health. Some studies have shown correlations between body weight gain and relative abundance of particular taxa, suggesting that the effects of stressors on the microbiota may contribute to observed changes in performance^11–13^. In one case, infection by *Eimeria tenella* decreased body weight gain, which was correlated to decreases in *Ruminococcus*, unclassified Lachnospiraceae, and *Lactobacillus* and an increase in unclassified CAB-I in the cecal mucosa^11^. In another study, ammonia decreased body weight gain, which was correlated to decreases in *Butyricicoccus*, *Parasutterella*, *Lachnospiraceae UCG-010*, *Ruminococcaceae UCG-013*, and *Ruminococcaceae UCG-004* and an increase in *Escherichia coli* in the cecal lumen^13^. To improve the performance of broilers under stressful conditions, microbiota-altering solutions such as probiotics, prebiotics, organic acids, essential oils, and polyphenols have been researched^14–18^.

Better understanding of the effects of HS on GIT bacterial composition and function is required to inform further research on microbiota modulation and dietary supplementation. The consequences of genetic selection on the broiler GIT microbiota are also unknown, requiring understanding of how the microbiota may respond to stressors differently in modern fast-growing broilers compared to ancestral slow-growing broilers. A previous study demonstrated that HS affects community composition of ileal microbiota, with more prominent effects on ileal mucosa compared to ileal luminal content^19^. Furthermore, analysis of the broiler genetic line showed that predicted community function could differ between genetic lines, with the largest shift in community function observed in the 2015 Modern Random Bred line compared to an ancestor, Giant Jungle Fowl. As the cecal microbiota is known to contain high absolute counts and diversity of bacteria^20^, in addition to being different to the ileal microbiota^21^, characterization of the cecal microbiota in HS birds may be valuable for devising nutritional strategies that maintain GIT microbial balance. The aim of this study was, therefore, to evaluate the effect of HS and genetic selection on the cecal luminal and mucosal microbiota of four genetic lines of broiler chickens.

## Materials and methods

### Animals and tissue sampling

The study was conducted in accordance with the recommendations in the guide for the care and use of laboratory animals of the National Institutes of Health and the protocols were approved by the University of Arkansas Animal Care and Use Committee under protocols 18,083 and 16,084. This study was performed and reported in accordance with ARRIVE guidelines (https://arriveguidelines.org/). Four lines of chicken were utilized for this study: Giant Jungle Fowl (JF), Athens Canadian Random Bred (ACRB), 1995 Arkansas Random Bred (L1995), and Modern Random Bred (L2015). Detailed characteristics of the lines were previously reported in Tabler et al.^3^. Protocols for incubation of embryonated eggs, hatching, housing conditions, and experimental design were as reported in Emami et al.^19^. In brief, to investigate the effect of heat stress (HS) on gut microbiota, chicks were separated by line and placed into twelve environmental chambers (24 pens total, 6 pens/line, 25 birds/pen, 0.09 m^2^/bird). Chamber temperatures were 32 °C at day 1 and gradually decreased to 20 °C at day 21. Water and 3-phase standard diets were provided ad libitum. At day 29, half of the pens for each line (3 pens/line) were raised under thermoneutral (TN, 24 °C) conditions, while 3 pens per line were subjected to chronic cyclic HS (8 h/day, 36 °C from 9 AM to 5 PM). On day 56, two birds per pen were selected based on the average pen weight and euthanized via cervical dislocation. The cecal contents (luminal, CeL) and cecal epithelial scrapings (mucosal, CeM) were collected for bacterial DNA sequencing.

### DNA isolation, library preparation, and sequencing

DNA isolation, library preparation, and sequencing were as reported in Emami et al.^19^. A PowerSoil kit (Qiagen, Valencia, CA), PCR primers targeting the V3–V4 region of the 16S rRNA gene, and the Illumina MiSeq platform (Illumina, Inc) were utilized for these steps, respectively. The 16S rRNA gene sequences determined in this study were deposited in the NCBI Sequence Read Archive database (SRA accession no. PRJNA930873).

### Bioinformatics and statistical analysis

Microbiota analyses on CeL and CeM microbiota were performed using the bioinformatics platform Quantitative Insights into Microbial Ecology 2 (QIIME 2) version 2022.8^22^. Quality control was performed through denoising with DADA2^23^ via the q2-dada2 plugin, setting truncation parameters using a quality cutoff of 30. The SILVA rRNA database version 138 was utilized for taxonomic analyses by downloading reference sequences and taxonomy files pre-formatted for QIIME 2 (obtained at https://docs.qiime2.org/2022.8/data-resources/) using RESCRIPt, a process that removes duplicate sequences assigned to different taxonomies to reduce inconsistencies and improve processing^24^. Reads were extracted from the reference sequences using the forward and reverse primers for the V3–V4 region, and the extracted reads were used to create a feature classifier via q2-feature-classifier^25^. Taxonomy was assigned to amplicon sequence variants (ASVs) from DADA2 via the q2-feature-classifier classify-sklearn naïve Bayes taxonomy classifier. Mitochondria, chloroplasts, and unassigned bacteria were filtered and excluded from the feature table. To construct a phylogeny, ASVs were aligned with MAFFT^26^ via q2-alignment and passed to fasttree2^27^ via q2-phylogeny. Rarefaction, or subsampling without replacement, was performed with sampling depths of 17,055 for CeL and 14,247 for CeM for alpha and beta diversity analyses via q2-diversity. Sampling depths were determined based on diversity captured at different depths (visualized by alpha rarefaction plots produced via q2-diversity) and the number of samples retained in the subset.

Alpha diversity metrics measure species richness and/or evenness within one sample. Shannon diversity index, observed features (ASVs), Faith’s phylogenetic diversity (Faith PD)^28^, and evenness were the alpha diversity metrics measured, and differences in alpha diversity between the TN and HS groups of the four lines were analyzed using the non-parametric Kruskal–Wallis test. Beta diversity metrics, in particular unweighted and weighted UniFrac distance^29^, were used to analyze similarity or dissimilarity between microbiota of samples while considering phylogeny. Presence and absence of ASVs in samples is considered by unweighted UniFrac analysis, while the abundance of ASVs is considered in weighted UniFrac analysis^30^. For statistical analysis of UniFrac distances, the non-parametric permutational analysis of variance (PERMANOVA) test was used. Principal coordinates analysis (PCoA) was used to visualize distances between microbiota, where clustering of points may indicate similarities or differences between microbiota within treatment groups. PCoA results from QIIME 2 were imported to R 4.1.2^31^ using the package QIIME2R 0.99.35^32^. Within the tidyverse 1.3.0^33^ package, dplyr was used to select PCoA axes (PC1 and PC2) and join metadata, and ggplot2 was used to produce alpha diversity box plots and PCoA scatter plots.

The linear discriminant analysis effect size (LEfSe) algorithm^34^ was used to analyze differential abundance of taxa up to the genus level between TN and HS birds in each line. The Huttenhower Lab Galaxy web server^35^ was used to perform LEfSe analyses, using the default parameters. Phylogenetic Investigation of Communities by Reconstruction of Unobserved States 2 (PICRUSt2) version 2.4.2 software^36^ was used to predict functional abundances based on marker gene sequences using the MetaCyc Metabolic Pathways Database^37^. PICRUSt2 output counts were transformed with center log-ratio transformation and inputted to STAMP 2.1.3^38^ to analyze and visualize predicted functional differences between TN and HS birds for each line, as well as compare all birds of the L2015 and JF lines.

### Ethics approval and consent to participate

The study was conducted in accordance with the recommendations in the guide for the care and use of laboratory animals of the National Institutes of Health and the protocols were approved by the University of Arkansas Animal Care and Use Committee under protocols 18083 and 16084. All methods were carried out in accordance with relevant guidelines and regulations. This study was performed and reported in accordance with ARRIVE guidelines (https://arriveguidelines.org/).

## Results

### Effects of heat stress on alpha diversity

**Figure 1:**
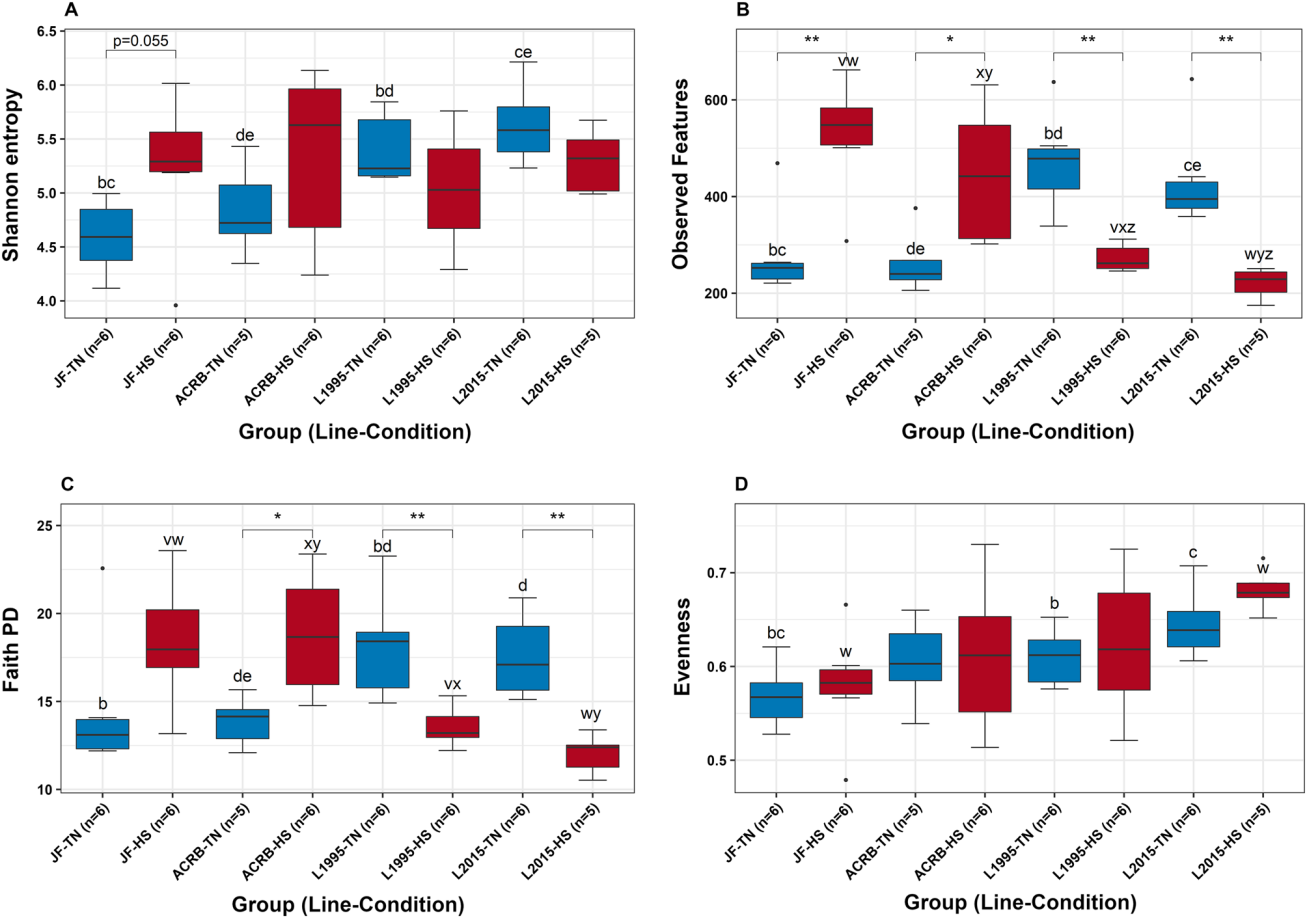
Comparisons of CeL microbiota alpha diversity: (**A**) Shannon diversity, (**B**) observed features (richness), (**C**) Faith’s Phylogenetic Diversity (Faith PD, richness), and (**D**) evenness. Stars denote statistically significant (*P < 0.05, **P < 0.01) differences. Letters a–f above boxes indicate significant differences between genetic lines of TN groups (a = JF and ACRB, b = JF and L1995, c = JF and L2015, d = ACRB and L1995, e = ACRB and L2015, f = L1995 and L2015). Letters u–z above boxes indicate significant differences between genetic lines of HS groups (u = JF and ACRB, v = JF and L1995, w = JF and L2015, x = ACRB and L1995, y = ACRB and L2015, z = L1995 and L2015).

Sequencing summaries of the CeL and CeM datasets are presented in Table 1. In CeL microbiota, Shannon diversity was significantly different based on groups (temperature condition and genetic line) overall (Kruskal–Wallis, H = 15.47, *P* = 0.03, Fig. 1A, and Additional file 1: Table S1), however, in pairwise comparisons between TN and HS groups for each genetic line, there were no significant differences (*P* > 0.05), though there was a trend of difference between TN and HS groups for the JF line (*P* = 0.055). Observed features were significantly different based on group (H = 39.81, *P* < 0.01, Fig. 1B, and Additional file 1: Table S2), including significantly lower observed features in HS birds compared to TN birds in the JF (H = 7.41, *P* < 0.01) and ACRB (H = 4.80, *P* = 0.03) lines and significantly higher observed features in HS birds in L1995 (H = 8.31, *P* < 0.01) and L2015 (H = 7.50, *P* < 0.01). Faith PD was significantly different based on group (H = 26.04, *P* < 0.01, Fig. 1C, and Additional file 1: Table S3), with significantly lower Faith PD in HS birds compared to TN birds in the ACRB line (H = 5.63, *P* = 0.02) and significantly higher Faith PD in HS birds in L1995 (H = 6.56, *P* = 0.01) and L2015 (H = 7.50, *P* < 0.01). There was only a trend of lower Faith PD in the JF line under HS (*P* = 0.08). Evenness significantly differed based on group (H = 15.04, *P* = 0.04, Fig. 1D, and Additional file 1: Table S4), however, there were no significant differences (*P* > 0.05) in comparisons between the TN and HS groups for all genetic lines, though there was a trend of higher evenness in the 2015 HS group compared to TN (*P* = 0.07). In CeM microbiota, there were no significant differences (*P* > 0.05, Additional file 1: Table S5) for all four alpha diversity metrics based on group.

**Table 1:** Sequencing summary of CeL and CeM microbiota datasets processed in QIIME 2. *QC* quality control via DADA2, *ASVs* amplicon sequence variants. Reads after filtering indicates the number of reads after exclusion of mitochondria, chloroplasts, and unassigned bacteria.

	CeL	CeM
Number of samples	48	48
Raw reads	8,709,198	7,222,122
Reads after QC	5,931,234	5,071,245
Reads after filtering	5,931,129	5,069,830
Reads per sample (range)	5272–862,135	7655–1,265,572
Mean reads per sample	123,565	105,621
Rarefaction sampling depth	17,055	14,247
Samples after rarefaction	46	40
Total number of ASVs	2363	2351
ASV read length (range)	284–487	282–513
Mean ASV read length	423	420

### Differences in alpha diversity in genetic lines

Pairwise comparisons were utilized where alpha diversity was significantly different based on groups to compare different genetic lines in the same temperature conditions. In CeL microbiota, Shannon diversity of the JF-TN group was lower compared to L1995-TN (H = 8.31, *P* < 0.01) and L2015-TN (H = 8.31, *P* < 0.01) and did not differ from ACRB-TN (*P* > 0.05, Fig. 1A, and Additional file 1: Table S1). Likewise, the ACRB-TN group was lower compared to L1995-TN (H = 4.03, *P* = 0.04) and L2015-TN (H = 5.63, *P* = 0.02). L1995-TN and L2015-TN did not differ in Shannon diversity (*P* > 0.05). Shannon diversity did not differ between genetic line under HS conditions (*P* > 0.05). Identical patterns were found in comparisons of TN groups for observed features and Faith PD (all *P* < 0.05, Fig. 1B,C, and Additional file 1: Tables S2 and S3), except for Faith PD only trending to be lower in JF-TN compared to L2015-TN (*P* = 0.055). JF-HS had greater observed features than L1995-HS and L2015-HS (both *P* < 0.05, Fig. 1B, and Additional file 1: Table S2), but it did not differ from ACRB-HS in observed features (*P* > 0.05). Observed features were different (*P* < 0.05) in all comparisons between ACRB-HS, L1995-HS, and L2015-HS, with observed features being lowest in L2015-HS and highest in ACRB-HS (Fig. 1B). JF-HS and ACRB-HS both had greater Faith PD than L1995-HS and L2015 HS (all *P* < 0.05), but Faith PD did not differ between JF-HS and ACRB-HS (*P* > 0.05, Fig. 1C, and Additional file 1: Table S3). Faith PD did not differ between L1995-HS and L2015-HS (*P* > 0.05). Evenness was lower in JF-TN compared to L1995-TN and L2015-TN (both *P* < 0.05, Fig. 1D, and Additional file 1: Table S4) and did not differ from ACRB-TN (*P* > 0.05). ACRB-TN, L1995-TN, and L2015-TN did not differ in evenness from each other in all comparisons (*P* > 0.05). Evenness was lower in JF-HS compared to L2015-HS (H = 6.53, *P* = 0.01), and there were no other significant comparisons between HS groups (all *P* > 0.05).

### Effects of heat stress on beta diversity

**Figure 2:**
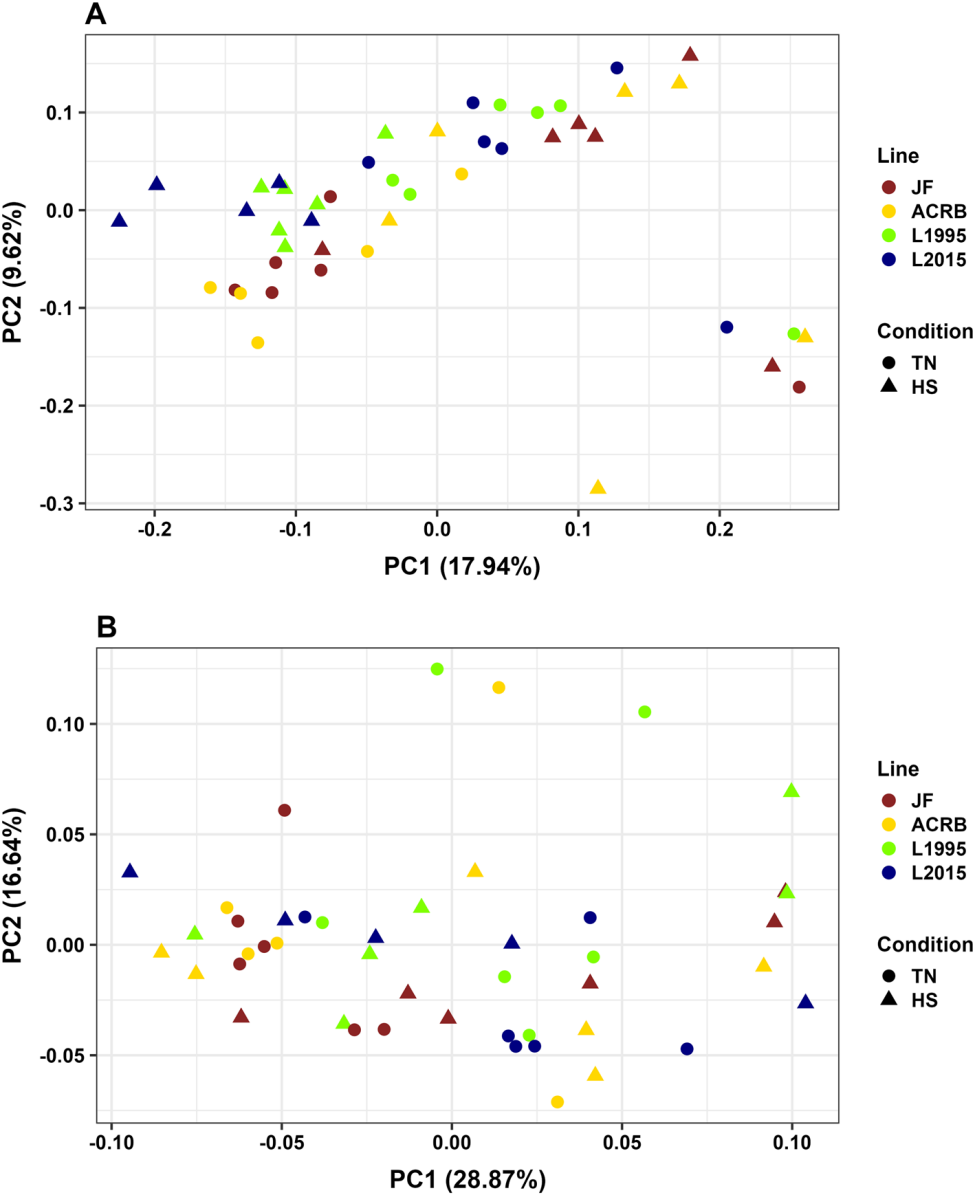
Principal coordinate analysis (PCoA) of CeL microbiota based on: (**A**) unweighted UniFrac and (**B**) weighted UniFrac. Colors indicate difference in lines, while shape of points indicate temperature condition. Longer distances between points indicate microbiota profiles were different, while shorter distances between points indicate profiles were similar.

CeL microbiota of different groups were distinct based on unweighted UniFrac distance (PERMANOVA, *P* < 0.01, visualized by PCoA in Fig. 2A, and statistics in Additional file 1: Table S6). This result included significant differences for comparisons between TN and HS birds for each genetic line: JF (*P* = 0.04), ACRB (*P* = 0.02), L1995 (*P* < 0.01), and L2015 (*P* < 0.01). When based on weighted UniFrac distance, microbiota of different groups were also distinct (*P* = 0.01, Fig. 2B, and Additional file 1: Table S7), however, only the JF line had significantly different microbiota (*P* = 0.02) between TN and HS birds. There were no significant differences (*P* > 0.05) between microbiota of TN and HS birds for the other three lines, though there was a trend of difference in the ACRB line (*P* = 0.06).

**Figure 3:**
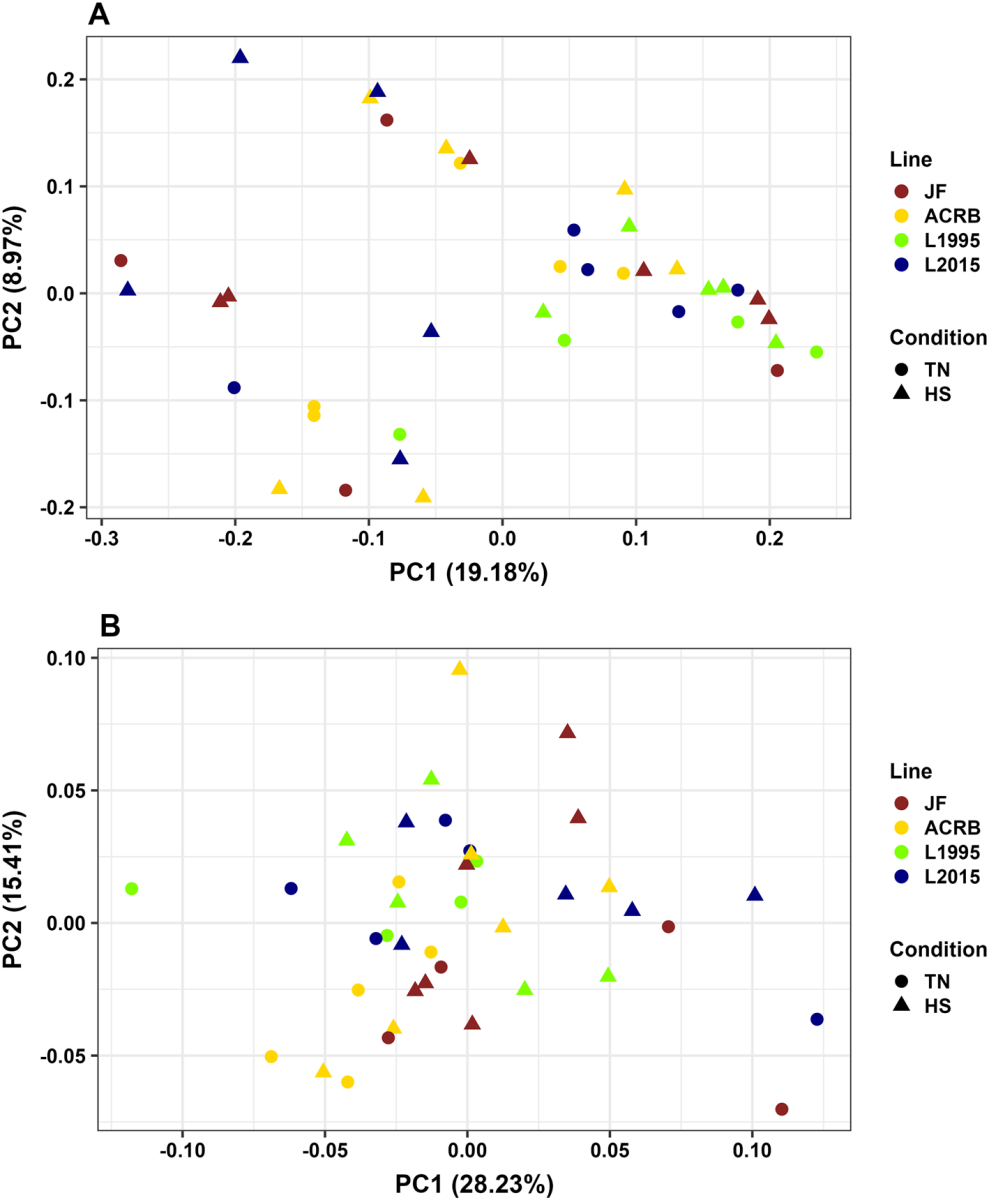
Principal coordinate analysis (PCoA) of CeM microbiota based on: (**A**) unweighted UniFrac and (**B**) weighted UniFrac. Colors indicate difference in lines, while shape of points indicate temperature condition. Longer distances between points indicate microbiota profiles were different, while shorter distances between points indicate profiles were similar.

CeM microbiota of different groups were distinct (*P* < 0.01, Fig. 3A, and Additional file 1: Table S8) based on unweighted UniFrac distance. In comparisons between TN and HS birds for each genetic line, there were significant differences in microbiota for L2015 birds (*P* = 0.04), but no significant differences for the JF, ACRB, or L1995 lines (*P* > 0.05). According to weighted UniFrac distance, there were differences in CeM microbiota in groups overall (*P* = 0.03, Fig. 3B, and Additional file 1: Table S9), however there were no significant differences (*P* > 0.05) in comparisons of microbiota between TN and HS birds for each genetic line.

### Beta diversity comparisons of genetic lines

Pairwise comparisons were utilized where beta diversity was significantly different based on groups to compare different genetic lines in the same temperature conditions. Based on unweighted UniFrac, the CeL microbiota of the JF-TN and ACRB-TN groups were distinct from L1995-TN and L2015-TN (all *P* < 0.05, Fig. 2A, and Additional file 1: Table S6), while JF-TN was not distinct from ACRB-TN and L1995-TN was not distinct from L2015-TN (both *P* > 0.05). ACRB-HS, L1995-HS, and L2015-HS were considered different to each other (all *P* < 0.05), while JF-HS was distinct from L1995-HS and L2015-HS, but not distinct from ACRB-HS. Based on weighted UniFrac, JF-TN were distinct from L1995-TN and L2015-TN (both *P* < 0.05, Fig. 2B, and Additional file 1: Table S7), but not from ACRB-TN (*P* > 0.05). ACRB-TN was distinct from L2015-TN (*P* = 0.03), but not from JF-TN or L1995-TN (*P* > 0.05), and L1995-TN and L2015-TN were not distinct (*P* > 0.05). Groups under HS conditions were not considered distinct from each other (all *P* > 0.05).

For CeM microbiota, JF-TN were not distinct from other TN groups based on unweighted UniFrac (all *P* > 0.05, Fig. 3A, and Additional file 1: Table S8). ACRB-TN was distinct from L1995-TN (*P* = 0.04), but not L2015-TN (*P* > 0.05), and L1995-TN and L2015-TN were not distinct (*P* > 0.05). JF-HS were not distinct from other HS groups (all *P* > 0.05), while ACRB-HS was distinct from L1995-HS (*P* = 0.01), but not distinct from L2015-HS (*P* > 0.05). L1995-HS was distinct from L2015-HS (*P* = 0.01). Based on weighted UniFrac, all groups under the TN condition were not distinct from each other, and all groups under the HS condition were not distinct from each other (all *P* > 0.05, Fig. 3B, and Additional file 1: Table S9).

### Differential bacterial abundance in heat-stressed and thermoneutral birds

**Figure 4:**
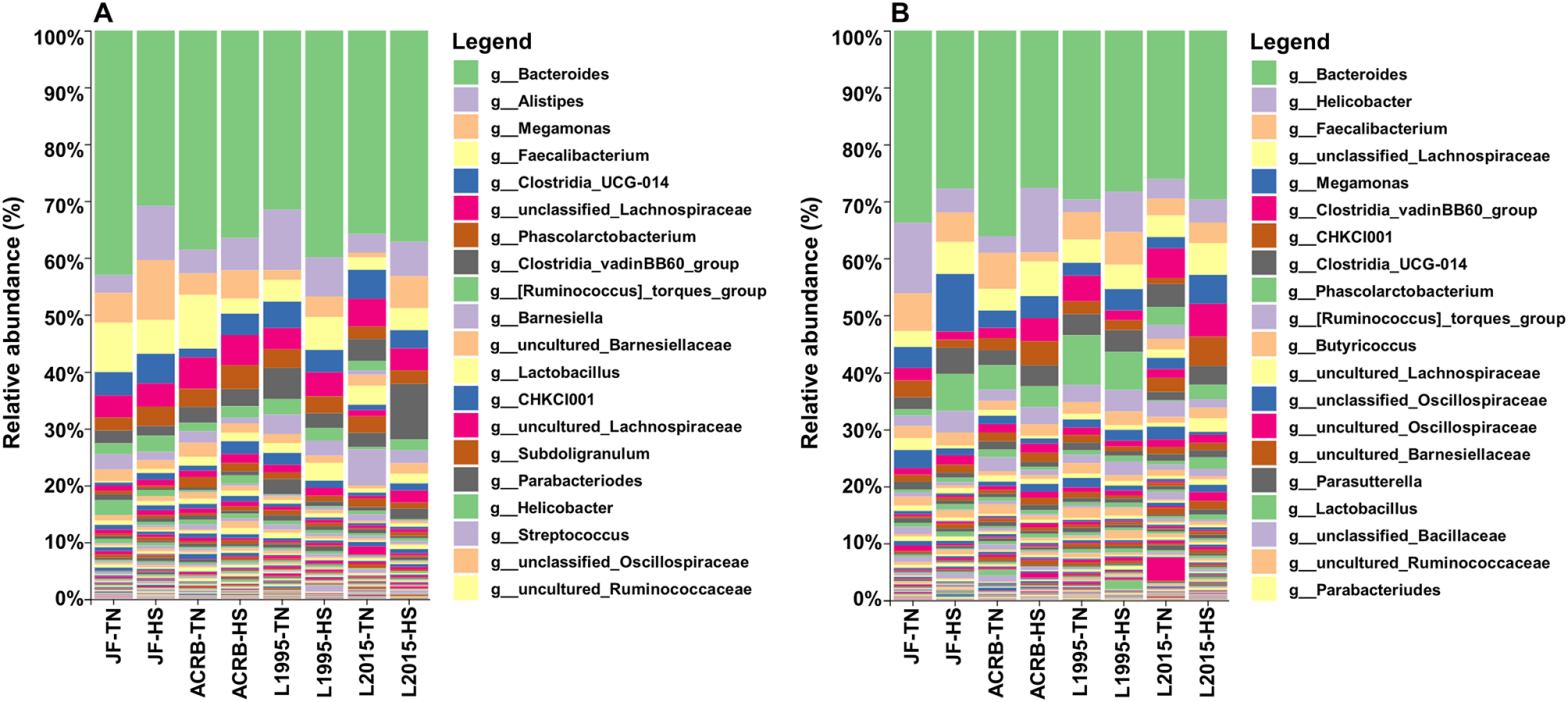
Relative abundances of bacterial taxa at the genus level in groups (line and condition; n = 6 samples for each column) of (**A**) CeL microbiota and (**B**) CeM microbiota. The 20 most abundant taxa overall are listed in the legend.

The bacterial profiles of CeL and CeM microbiota by group (line and condition) are displayed in Fig. 4A,B, respectively. In CeL microbiota, the top five genera present in all samples were *Bacteroides*, *Alistipes*, *Megamonas*, *Faecalibacterium*, and *Clostridia UCG-014* (Fig. 4A). In CeM microbiota, the top five genera present in all samples were *Bacteroides*, *Helicobacter*, *Faecalibacterium*, unclassified *Lachnospiraceae*, and *Megamonas* (Fig. 4B).

**Figure 5:**
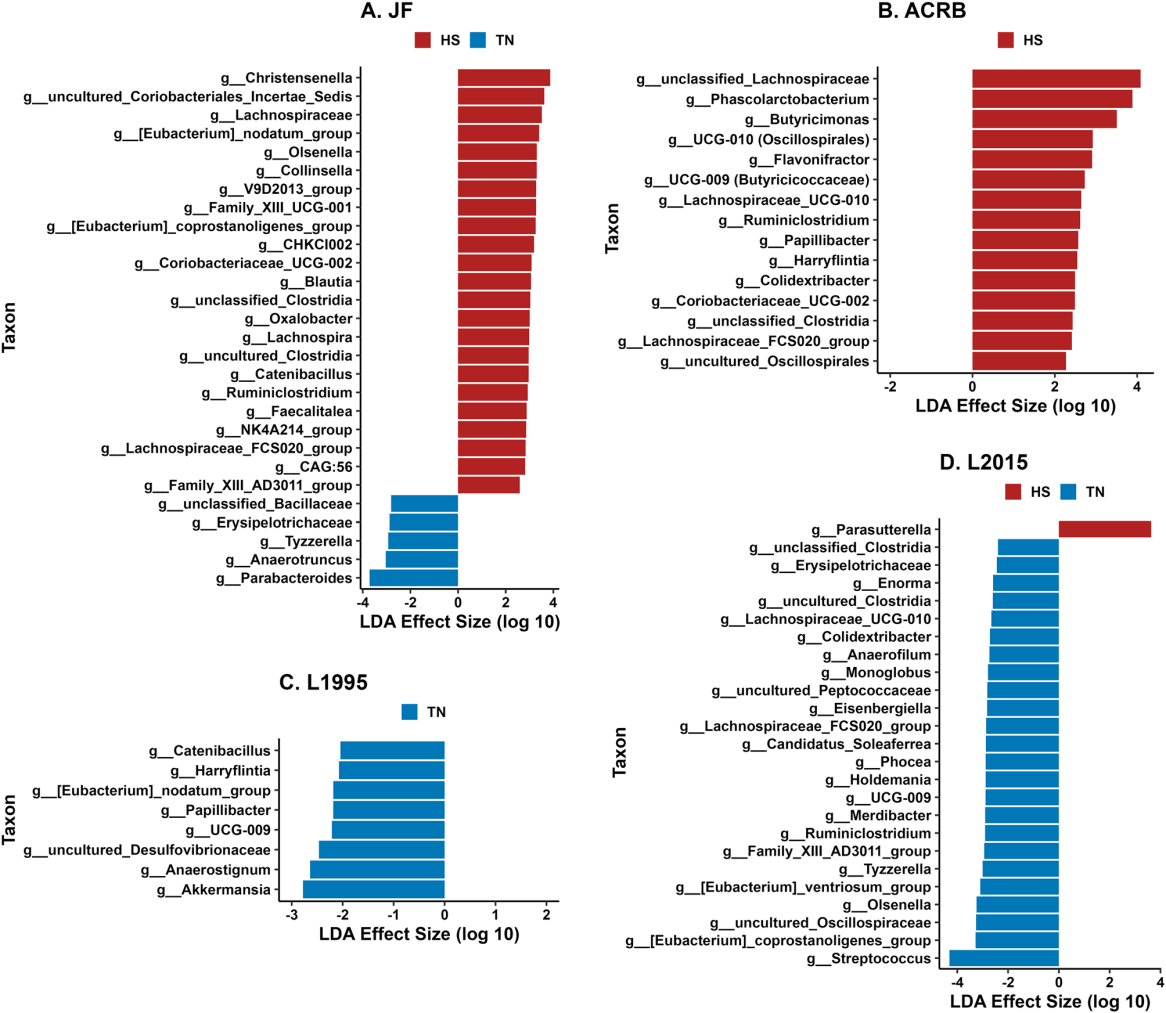
Linear discriminant analysis effect size (LEfSe) in comparisons of CeL microbiota in HS and TN groups for each line: (**A**) Jungle Fowl (JF), (**B**) Athens Canadian Random Bred (ACRB), (**C**) 1995 Random Bred (L1995), (**D**) 2015 Modern Random Bred (L2015). Positive effect size indicates higher relative abundance in HS group, while negative effect size indicates higher relative abundance in TN group.

In CeL microbiota of JF birds, 23 genera were in greater relative abundance in HS birds (Fig. 5A), including *Christensenella*, uncultured *Coriobacteriales Incertae Sedis*, *Lachnospiraceae*, [*Eubacterium*] *nodatum group* (brackets indicate contested names in SILVA database), and *Olsenella*. Five genera had greater relative abundance in TN birds: *Parabacteroides*, *Anaerotruncus*, *Tyzzerella*, *Erysipelotrichaceae*, and unclassified *Bacillaceae*. In ACRB birds, 15 genera were in greater relative abundance in HS birds compared to TN birds (Fig. 5B). The 5 genera with the greatest effect size were unclassified *Lachnospiraceae*, *Phascolarctobacterium*, *Butyricimonas*, *UCG-010* (Oscillospirales), and *Flavonifractor*. In L1995 birds, 8 genera were in greater relative abundance in TS birds (Fig. 5C), including *Akkermansia*, *Anaerostignum*, uncultured *Desulfovibrionaceae*, *UCG-009*, and *Papillibacter*. In L2015 birds, *Parasutterella* was in greater relative abundance in HS birds, while 24 genera were in greater relative abundance in TS birds, including *Streptococcus*, [*Eubacterium*] *coprostanoligenes group*, uncultured *Oscillospiraceae*, *Olsenella*, and [*Eubacterium*] *ventriosum group* (Fig. 5D).

**Figure 6:**
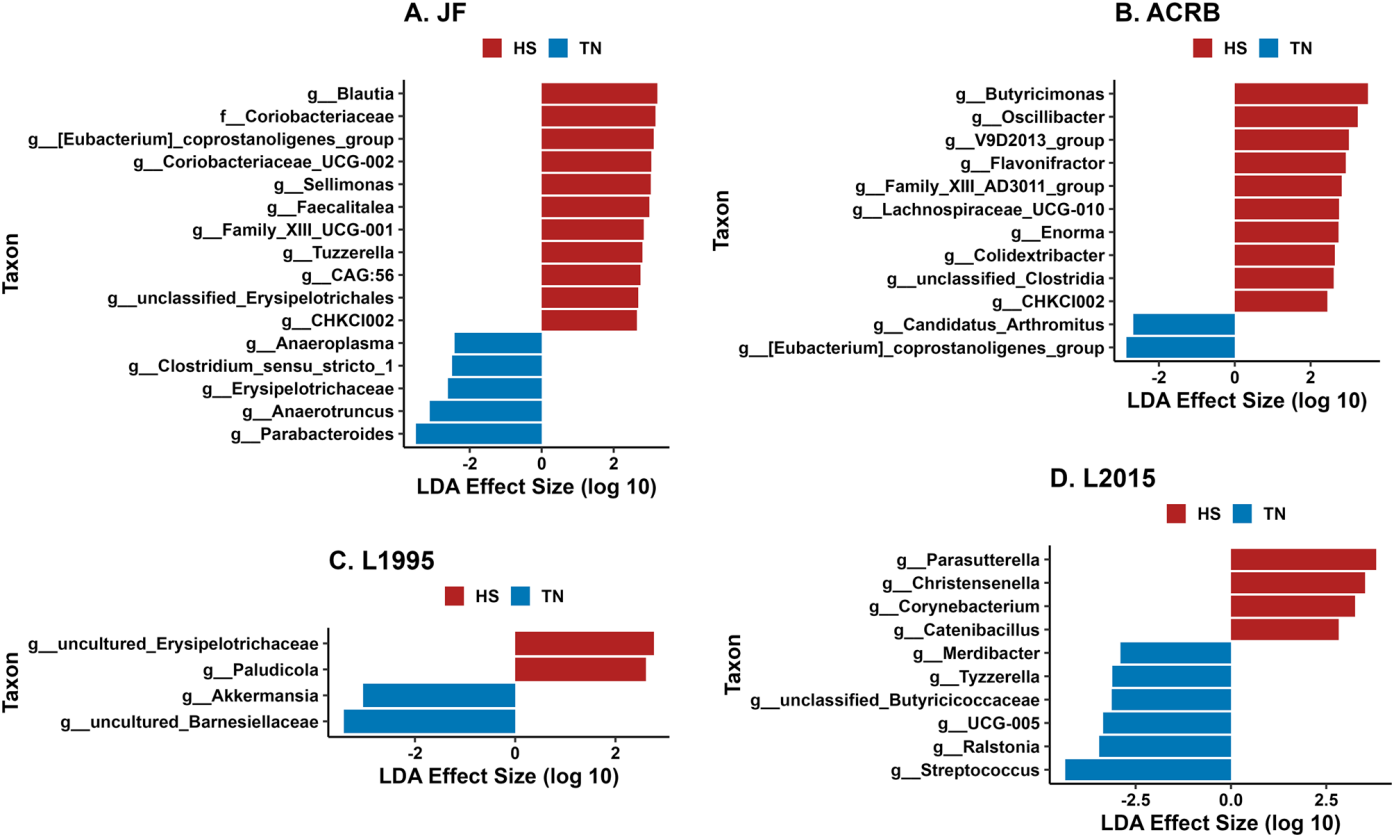
Linear discriminant analysis effect size (LEfSe) in comparisons of CeM microbiota in HS and TN groups for each line: (**A**) Jungle Fowl (JF), (**B**) Athens Canadian Random Bred (ACRB), (**C**) 1995 Random Bred (L1995), (**D**) 2015 Modern Random Bred (L2015). Positive effect size indicates higher relative abundance in HS group, while negative effect size indicates higher relative abundance in TN group.

In the cecal mucosal microbiota of JF birds, 10 genera, including *Blautia*, [*Eubacterium*] *coprostanoligenes group*, *Coriobacteriaceae UCG-002*, *Sellimonas*, and *Facalitalea*, and the family *Coriobacteriaceae*, were in greater relative abundance in HS birds, while *Parabacteroides*, *Anaerotruncus*, *Erysipelotrichaceae*, *Clostridium *sensu stricto* 1*, and *Anaeroplasma* were in greater relative abundance in TN birds (Fig. 6A). In ACRB birds, 10 genera were in greater relative abundance in HS birds, including *Butyricimonas*, *Oscillibacter*, *V9D2013 group*, *Flavonifractor*, and *Family XIII AD3011 group*, while *Candidatus Arthromitus* and [*Eubacterium*] *coprostanoligenes group* were in greater relative abundance in TN birds (Fig. 6B). In L1995 birds, uncultured Erysipelotrichaceae and *Paludicola* were in greater relative abundance in HS birds, while uncultured *Barnesiellaceae* and *Akkermansia* were in greater relative abundance in TS birds (Fig. 6C). In L2015 birds, *Parasutterella*, *Christensenella*, *Corynebacterium*, and *Catenibacillus* were in greater relative abundance in HS birds, while 6 genera, including *Streptococcus*, *Ralstonia*, *UCG-005*, unclassified *Butyricoccaceae*, and *Tyzzerella* were in greater relative abundance in TN birds (Fig. 6D).

### Predicted functional abundances

**Figure 7:**
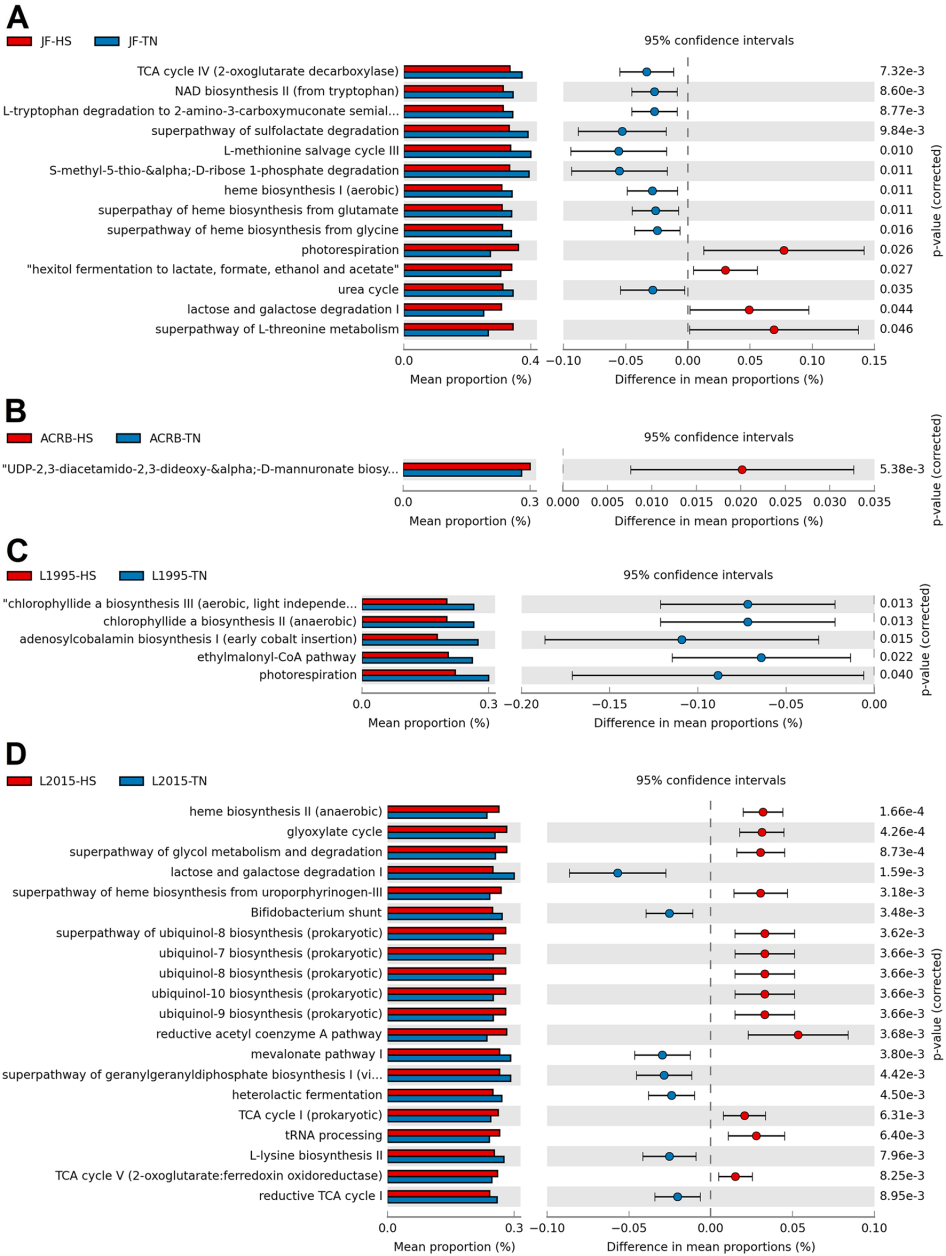

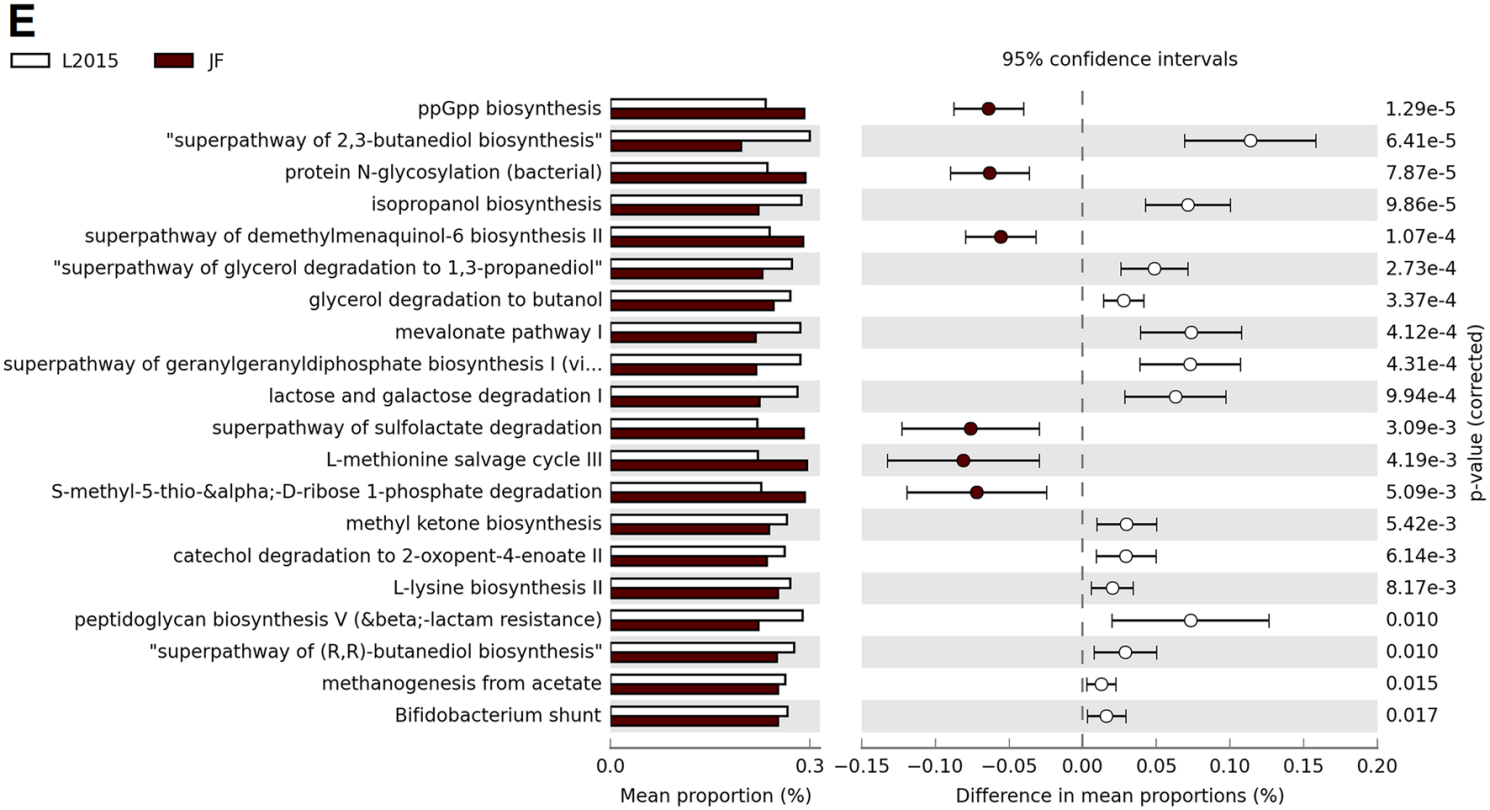
Effect of HS on the mean proportion (%) of predicted MetaCyc pathways (up to the top 21 shown) in the CeL microbiota of each line: (**A**) Jungle Fowl (JF), (**B**) Athens Canadian Random Bred (ACRB), (**C**) 1995 Random Bred (L1995), (**D**) 2015 Modern Random Bred (L2015). In (**E**), L2015 and JF were compared to investigate the differences in a modern line and an ancestral line.

In CeL microbiota of JF, HS decreased the relative abundance of genes for 10 predicted MetaCyc pathways compared to TN birds, while HS increased relative abundance of 4 pathways (Fig. 7A). Decreased pathways included TCA cycle IV (2-oxoglutarate decarboxylase), NAD biosynthesis II (from tryptophan), l-tryptophan degradation to 2-amino-3-carboxymuconate semialdehyde, superpathway of sulfolactate degradation, and l-methionine salvage cycle III. Increased pathways included photorespiration, “hexitol fermentation to lactate, formate, ethanol and acetate”, lactose and galactose degradation I, and superpathway of l-threonine metabolism. In ACRB, HS increased the relative abundance of the pathway UDP-2,3-diacetamido-2,3-dideoxy-α-d-mannuronate biosynthesis (Fig. 7B). In L1995, HS decreased the relative abundance of 5 pathways, including chlorophyllide a biosynthesis III (aerobic, light independent), chlorophyllide a biosynthesis II (anaerobic), adenosylcobalamin biosynthesis I (early cobalt insertion), ethylmalonyl-CoA pathway, and photorespiration (Fig. 7C). In L2015, HS decreased the relative abundance of 9 pathways and increased the relative abundance of 31 pathways (top 20 results by *P*-value displayed in Fig. 7D). Decreased pathways included lactose and galactose degradation I, Bifidobacterium shunt, mevalonate pathway I, superpathway of geranylgeranyldiphosphate biosynthesis I (via mevalonate), and heterolactic fermentation. Increased pathways included those involved in ubiquinol biosynthesis (ubiquinol-7, -8, -9, and -10) and heme biosynthesis (II and superpathway).

**Figure 8:**
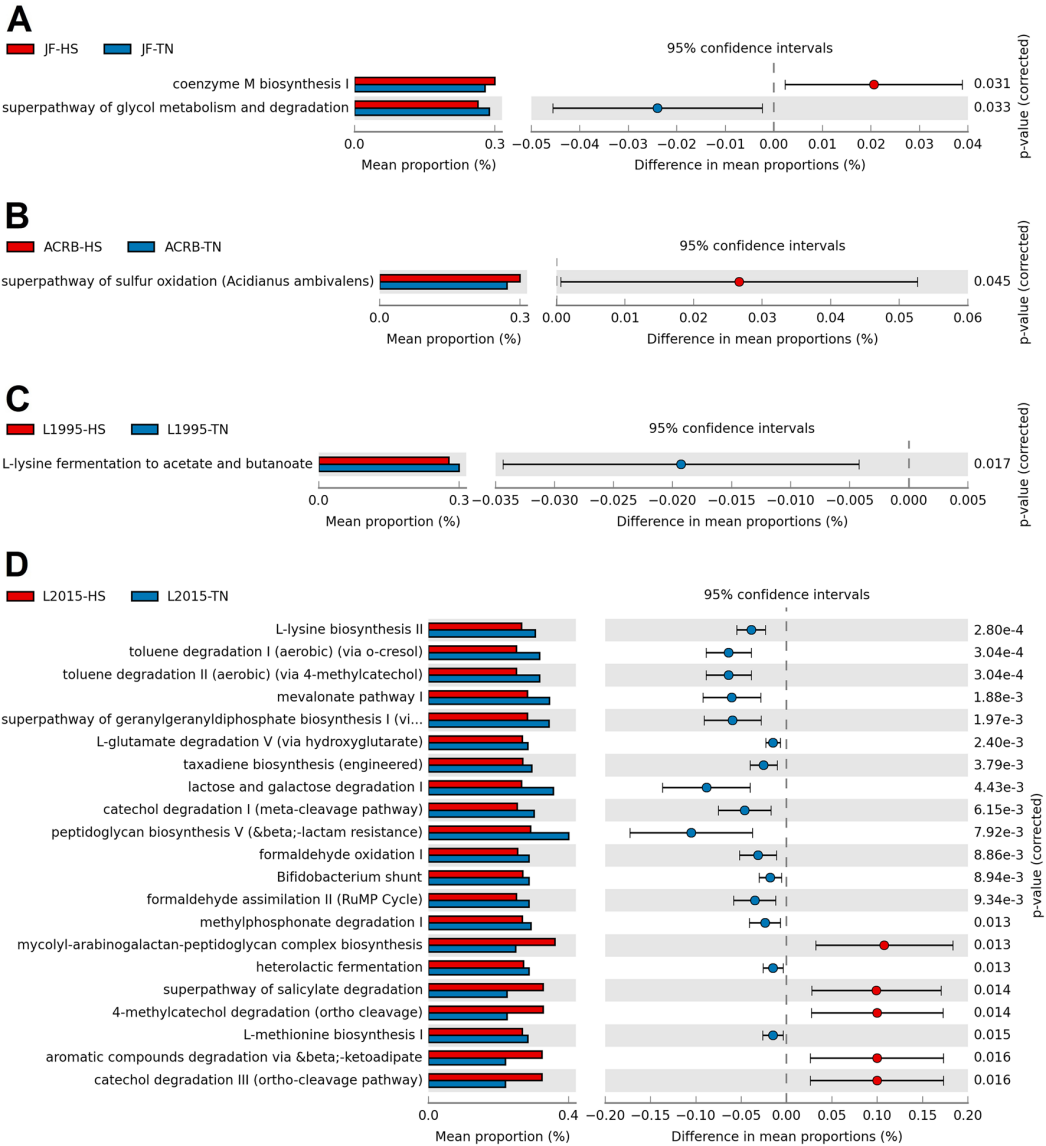

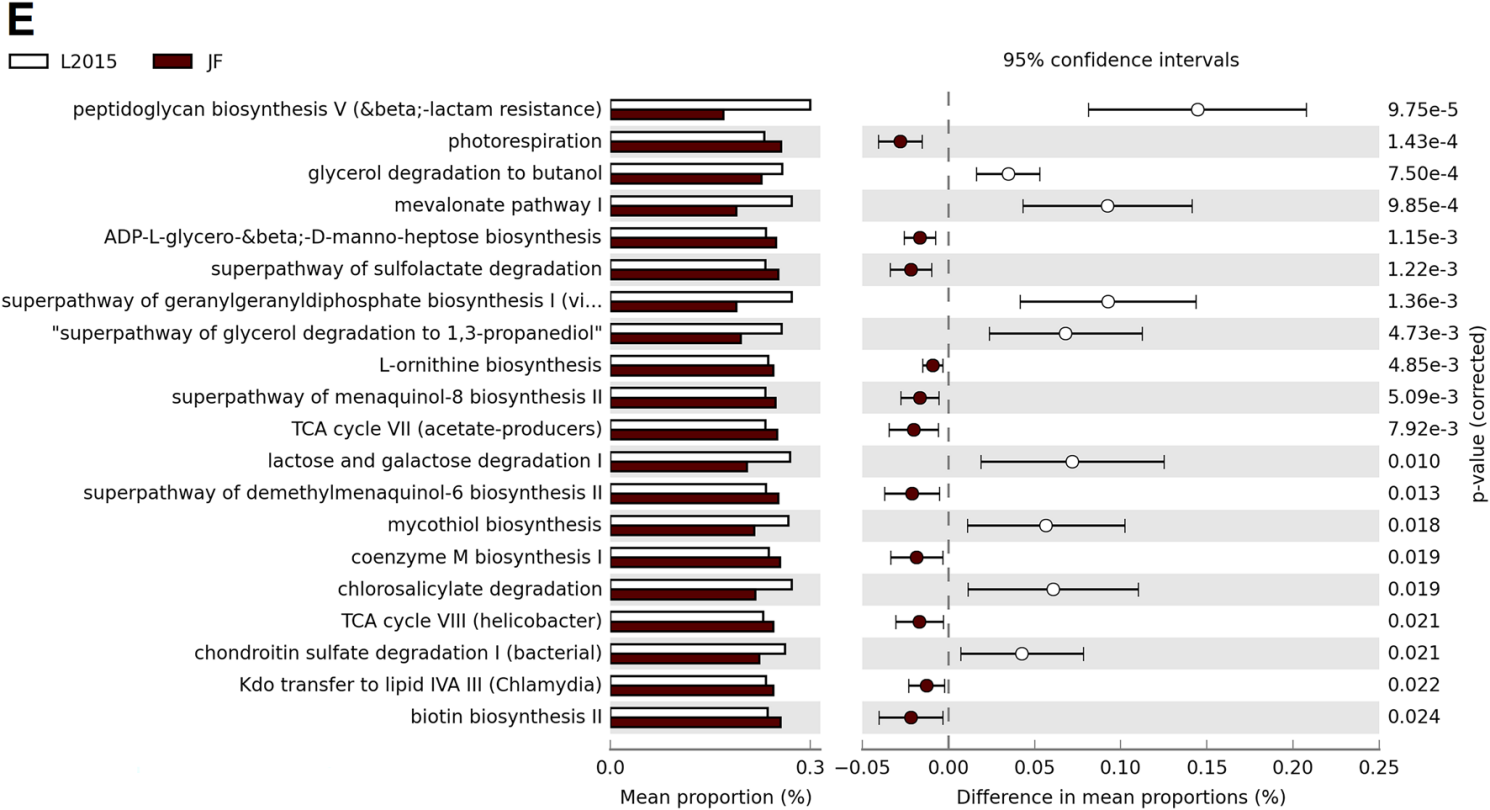
Effect of HS on the mean proportion (%) of predicted MetaCyc pathways (up to the top 21 shown) in the CeM microbiota of each line: (**A**) Jungle Fowl (JF), (**B**) Athens Canadian Random Bred (ACRB), (**C**) 1995 Random Bred (L1995), (**D**) 2015 Modern Random Bred (L2015). In (**E**), L2015 and JF were compared to investigate the differences in a modern line and an ancestral line.

In CeM microbiota of JF, HS decreased the relative abundance of the superpathway of glycol metabolism and degradation, while it increased coenzyme M biosynthesis I (Fig. 8A). In ACRB, HS increased the relative abundance of the superpathway of sulfur oxidation (*Acidianus ambivalens*) (Fig. 8B). In L1995, HS decreased the relative abundance of the pathway l-lysine fermentation to acetate and butanoate (Fig. 8C). In L2015, HS decreased the relative abundance of 22 pathways and increased the relative abundance of 22 pathways (top 21 results by *P*-value displayed in Fig. 8D). Decreased pathways included l-lysine biosynthesis II, toluene degradation I and II, mevalonate pathway I, and superpathway of geranylgeranyldiphosphate biosynthesis I (via mevalonate). Increased pathways included mycolyl-arabinogalactan-peptidoglycan complex biosynthesis, superpathway of salicylate degradation, 4-methylcatechol degradation (ortho cleavage), aromatic compounds degradation via beta-ketoadipate, and catechol degradation III (ortho cleavage).

All L2015 birds were compared with JF birds to compare predicted functional abundance between a modern line and an ancestral line. In CeL microbiota, 18 pathways were in greater relative abundance in L2015 birds, and 13 pathways were in greater relative abundance in JF birds (Fig. 7E). Pathways in greater abundance in L2015 included ppGpp biosynthesis, protein N-glycosylation (bacterial), superpathway of demethylmenaquinol-6 biosynthesis II, superpathway of sulfolactate degradation, and l-methionine salvage cycle III. Pathways in greater abundance in JF included superpathway of 2,3-butanediol biosynthesis, isopropanol biosynthesis, superpathway of glycerol degradation to 1,3-propanediol, glycerol degradation to butanol, and mevalonate pathway I. In CeM microbiota, 17 pathways were in greater relative abundance in L2015, and 22 pathways were in greater relative abundance in JF birds (Fig. 8E). Pathways in greater abundance in L2015 included peptidoglycan biosynthesis V (beta; -lactam resistance), glycerol degradation to butanol, mevalonate pathway I, superpathway of geranylgeranyldiphosphate biosynthesis I (via mevalonate), and superpathway of glycerol degradation to 1,3-propanediol.

## Discussion

The objectives of this study were to understand the effect of HS on factors such as alpha and beta diversity, bacterial abundance, and predicted metabolic function in the CeL and CeM microbiota, in addition to comparing these factors between four genetic lines of broilers and determining whether HS affected the microbiota differently in a line-dependent manner. Our findings demonstrate that HS had more prominent effects on the CeL microbiota compared to CeM and that the type of effects observed from HS were heavily dependent on genetic line. Lastly, predictive functional analysis showed that further studies in metabolic function of modern broilers may be of interest, as HS was predicted to have greater effects on metabolic pathways in modern L2015 compared to other lines. These findings are of importance in understanding both the roles of heat stress and host genetics on the microbiota, informing further research on development of effective microbiota-altering strategies (e.g. probiotics) to mitigate the negative effects of environmental stressors such as heat stress.

Our alpha diversity results showed that HS affected richness (observed features) in the CeL microbiota of all lines, however, the effects were in opposite directions in the slow-growing JF and ACRB lines compared to the moderate-growing L1995 and modern fast-growing L2015. Interestingly, richness increased in the two slow-growing lines, while it decreased in the two faster-growing lines. Results followed the same pattern for another richness metric, Faith PD, except for HS having only a trend of increasing Faith PD in the JF line. These results suggest that potential microbiota-altering solutions would need to consider differences in genetic lines, including physiological differences and bacterial composition. It has been previously shown that HS increases core body temperature in the more modern L1995 and L2015 lines, but not in the JF or ACRB lines^3^, which could be one explanation for the opposing effects, as bacteria would be responding to different temperature conditions. Alternatively, genetic differences in lines have been found to influence intestinal microbiota composition^39,40^, therefore the differences in composition could lead to different responses to HS and contribute to the differential changes in core temperature. Our data showed richness, as well as Shannon diversity, were significantly lower in the CeL microbiota of TN birds in the JF and ACRB lines compared to L1995 and L2015. These different starting points in diversity may influence competition dynamics of bacteria in the slower and faster-growing lines, affecting how richness changes during disruption by HS. Alpha diversity in CeM microbiota was not significantly affected by HS, and this result again demonstrates the differences between luminal and mucosal microbiota and the importance of understanding bacterial profiles of both regions, as they can be affected by stressors differently. Differing effects on luminal and mucosal microbiota were also observed in a previous experiment on the effect of HS on ileal microbiota, where we found HS had more prominent effects on Shannon diversity and taxonomic abundance in the ileal mucosa compared to the lumen^19^.

Beta diversity results supported alpha diversity findings showing that HS primarily altered CeL microbiota. Specifically, bacterial profiles tended to be affected by the presence and absence of bacterial taxa (unweighted UniFrac) as opposed to the abundances of bacterial taxa (weighted UniFrac). In all four lines, CeL microbiota were considered distinct between TN and HS birds in unweighted UniFrac analysis, while weighted UniFrac analysis determined CeL microbiota differed in HS birds only in the JF line. This was consistent with the alpha diversity results, as richness (presence and absence) was affected by HS. From visualization by PCoA, a puzzling phenomenon is observed where CeL bacterial profiles of faster-growing L1995 and L2015 birds under HS became more similar to TN bacterial profiles of slower-growing JF and ACRB birds. Meanwhile, CeL bacterial profiles of JF and ACRB birds under HS became more similar to TN bacterial profiles of L1995 and L2015 birds. CeM microbiota again displayed different patterns, where TN and HS microbiota differed only in L2015 birds, based on unweighted UniFrac. Future research to understand the underlying physiological and functional differences in different genetic lines may be of importance in developing probiotics or supplementation solutions that are suitable for the microbiota of specific breeds.

Although bacterial profiles primarily differed from presence and absence of taxa rather than abundance under HS, we could still analyze differential abundance in particular bacterial taxa at the genus level. Similar to how HS appeared to result in decreased richness in the CeL microbiota of more modern lines, HS appeared to decrease the relative abundance of certain genera in modern lines. Except for *Parasutterella* in L2015-HS birds, all other differentially abundant taxa were in greater relative abundance in the TN groups of the faster-growing L1995 and L2015 lines. In the slower-growing ACRB and JF lines, we observed the opposite pattern, where the majority of differentially abundant taxa were those that increased in relative abundance in the HS groups, while only five genera in JF birds were greater in the TN group. These results again demonstrate that genetic line plays a large role in how HS affects the CeL microbiota, as overall richness and abundance of certain taxa can be affected in opposite ways in different lines. In the L1995 and L2015 lines, genera within the orders *Lachnospirales* and *Oscillospirales*, which are typically obligate anaerobes, were the most likely to decrease in relative abundance from HS. The intestinal lumen is anoxic under healthy conditions, however, the oxidative stress that can occur during HS can affect the oxygen gradient, shifting the microbiota balance from obligate anaerobes to facultative anaerobes^41^. This could explain the decrease of these genera in HS birds of L1995 and L2015, though the dynamics may differ in JF and ACRB. Dynamics such as these may make certain groups of taxa more appropriate for inclusion in microbiota-altering solutions, or indicate the need to supplement metabolites to account for the reduced abundance of taxa which may be responsible in producing those metabolites.

While decreases in genera were the norm in L2015, *Parasutterella* was significantly higher under HS in both CeL and CeM microbiota of L2015, similar to results in another study on ileal mucosal microbiota of the same line^19^. *Parasutterella* is an obligate anaerobe of the phylum *Proteobacteria*, and potentially plays a role in bile acid maintenance and cholesterol metabolism due to its correlations with microbial-derived metabolites such as aromatic amino acid, bilirubin, purine, and bile acid derivatives^42^. Unlike our studies, *Parasutterella* has been positively correlated with performance in the CeL microbiota^13^, though this occurred due to ammonia exposure as opposed to HS. While HS tended to decrease relative abundance of other genera in CeL microbiota of L2015 birds, *Parasutterella* was the only genus in greater relative abundance, possibly indicating a unique interaction under HS. In contrast, *Streptococcus* and *Tyzzerella* decreased in relative abundance under HS in both the CeL and CeM microbiota of L2015 birds, and the same was true for *Akkermansia* in L1995 birds. *Streptococcus* has also been seen to decrease in the ileal microbiota of Arbor Acres broilers^6^ and has been negatively correlated with broiler body weight in the ileum^12^, however, further investigation is required as some *Streptococcus* species may function as normal gut flora while others cause disease. In the same study, *Akkermansia* was found to negatively correlate with body weight in both the cecum and ileum^12^, however, the abundance of *Akkermansia* in our study was generally low and unlikely to have a significant functional impact. Unlike in the ileal mucosa of our previous study and in the CeL of Arbor Acres broilers in Liu et al.^43^, *Tyzzerella* instead decreased under HS. This genus has been linked to cardiovascular disease in humans^44^.

As relative abundance of taxa may be affected by HS, future studies may seek to link changes in abundance of taxa to changes in metabolic function. To predict metabolic functional abundances, we utilized functional analysis. Although accuracy of functional analysis can be limited outside of human studies^45^, the tool provides a cost-free method of predicting functional abundances from 16S rRNA sequencing, which may assist in informing further research utilizing shotgun metagenome sequencing. Function of the gut microbiota is hypothesized to differ in modern broilers compared to ancestral broilers, as function is a potential link between the differences in bacterial composition in broiler lines observed in this study and the greater susceptibility of modern broilers to HS reported previously^3^. Our results from comparison of predicted function in CeL microbiota showed genes predicted to influence ppGpp biosynthesis were in greater relative abundance in the JF line. ppGpp biosynthesis is involved in the bacterial stringent response to environmental stresses, where a switch in transcription profile occurs to rapidly produce factors necessary for stress resistance, glycolysis, and amino acid synthesis^46^. Other pathways more frequent in JF included protein N-glycosylation and l-methionine salvage cycle III, which may indicate the CeL microbiota of the JF line has a strong response to environmental stressors. Multiple pathways related to production of alcohols, such as butanediol, isopropanol, and butanol, were in greater abundance in both the CeL and CeM microbiota of L2015 birds.

In addition to comparison of L2015 and JF birds, HS and TN birds were compared within each line. Overall, HS had more prominent effects on the predicted functional abundances in L2015 birds in both the CeL and CeM microbiota. Very few changes occurred in ACRB and L1995 birds, while there were some changes in the CeL microbiota of JF birds such as decreased relative abundance of pathways related to heme biosynthesis, TCA cycle IV, and usage of amino acids like tryptophan and methionine. Numerous predicted pathways were differentially abundant in L2015 birds, totaling 40 pathways in CeL and 44 pathways in CeM. In CeL microbiota, multiple ubiquinol biosynthesis pathways were in increased abundance under HS, which may be a response to deal with overproduction of free radicals under stressful conditions^47^. This prediction was also observed in the CeM microbiota of *Eimeria tenella*-infected broilers^11^. Unlike in JF, pathways related to heme biosynthesis were increased under HS. Lactose and galactose degradation I was decreased under HS in both CeL and CeM, which may have implications as prebiotics often contain lactose, galactose, and/or related sugars^9^.

## Conclusions

HS affected the bacterial richness and resulted in distinct bacterial profiles in the cecal luminal microbiota in four genetic lines, decreasing richness in more modern lines and increasing richness in older lines. HS had limited effects in cecal mucosal microbiota, only resulting in distinct bacterial profiles in L2015. Differences in bacterial profiles in different genetic lines appeared to influence patterns of differential taxonomic abundance, potentially affecting likelihood of increases or decreases in certain taxa. Predicted functional abundances suggested that L2015 broilers may have functional differences to the ancestral JF line, and selection leading to modern L2015 broilers may have made modern broilers more susceptible to functional changes under HS. Continued research on GIT microbiota and the function of its members is of importance to alleviate the negative effects of HS on modern broiler chickens’ performance and health.

### Supplementary Information

Supplementary Information.

## Data Availability

The 16S rRNA gene sequences determined in this study were deposited in the NCBI Sequence Read Archive (SRA) database (https://www.ncbi.nlm.nih.gov/bioproject; SRA accession no. PRJNA930873).

## Abbreviations

ACRB: Athens-Canadian Random Bred
ASV: Amplicon sequence variant
CeL: Cecal luminal bacterial population
CeM: Cecal mucosal bacterial population
d: Day
GIT: Gastrointestinal tract
HS: Heat stress
JF: Giant Jungle Fowl
L1995: 1995 Random bred
L2015: Modern random bred
PCoA: Principal coordinate analysis
PERMANOVA: Non-parametric multivariate ANOVA
PICRUSt: Phylogenetic Investigation of Communities by Reconstruction of Unobserved States
QIIME: Quantitative Insights Into Microbial Ecology
SRA: NCBI sequence read archive
STAMP: Statistical analysis of metagenomic profiles
TN: Thermoneutral
