# Supplementary material for: Cecal microbiota composition differs under normal and high ambient temperatures in genetically distinct chicken lines
Source: Sci Rep. 2023 Sep 25;13:16037. doi: 10.1038/s41598-023-43123-9 (PMC10519933; doi:10.1038/s41598-023-43123-9)
Supplement: Supplementary file 1 — Supplementary Information. [file 41598_2023_43123_MOESM1_ESM.docx]

**Additional file 1**

**Table S1.** Comparison of Shannon diversity in the cecal luminal microbiota of birds from 4 genetic lines and 2 conditions (thermoneutral [TN] or heat stressed [HS]) using the Kruskal-Wallis test.

| **Kruskal-Wallis** | **H** | **p-value** |
| --- | --- | --- |
| All groups | 15.5 | 0.030 |

| **Group 1** | **Group 2** | **H** | **p-value** | **q-value** |
| --- | --- | --- | --- | --- |
| L1995-HS (n=6) | L1995-TN (n=6) | 2.56 | 0.109 | 0.349 |
|  | L2015-HS (n=5) | 0.53 | 0.465 | 0.592 |
|  | L2015-TN (n=6) | 3.69 | 0.055 | 0.219 |
|  | ACRB-HS (n=6) | 0.64 | 0.423 | 0.592 |
|  | ACRB-TN (n=5) | 0.30 | 0.584 | 0.681 |
|  | JF-HS (n=6) | 0.64 | 0.423 | 0.592 |
|  | JF-TN (n=6) | 1.64 | 0.200 | 0.376 |
| L1995-TN (n=6) | L2015-HS (n=5) | 0.30 | 0.584 | 0.681 |
|  | L2015-TN (n=6) | 2.08 | 0.150 | 0.349 |
|  | ACRB-HS (n=6) | 0.10 | 0.749 | 0.806 |
|  | ACRB-TN (n=5) | 4.03 | 0.045 | 0.219 |
|  | JF-HS (n=6) | 0.10 | 0.749 | 0.806 |
|  | JF-TN (n=6) | 8.31 | 0.004 | 0.055 |
| L2015-HS (n=5) | L2015-TN (n=6) | 2.13 | 0.144 | 0.349 |
|  | ACRB-HS (n=6) | 0.53 | 0.465 | 0.592 |
|  | ACRB-TN (n=5) | 2.45 | 0.117 | 0.349 |
|  | JF-HS (n=6) | 0.03 | 0.855 | 0.873 |
|  | JF-TN (n=6) | 6.53 | 0.011 | 0.099 |
| L2015-TN (n=6) | ACRB-HS (n=6) | 0.03 | 0.873 | 0.873 |
|  | ACRB-TN (n=5) | 5.63 | 0.018 | 0.123 |
|  | JF-HS (n=6) | 1.64 | 0.200 | 0.376 |
|  | JF-TN (n=6) | 8.31 | 0.004 | 0.055 |
| ACRB-HS (n=6) | ACRB-TN (n=5) | 1.20 | 0.273 | 0.450 |
|  | JF-HS (n=6) | 0.64 | 0.423 | 0.592 |
|  | JF-TN (n=6) | 2.08 | 0.150 | 0.349 |
| ACRB-TN (n=5) | JF-HS (n=6) | 1.63 | 0.201 | 0.376 |
|  | JF-TN (n=6) | 1.20 | 0.273 | 0.450 |
| JF-HS (n=6) | JF-TN (n=6) | 3.69 | 0.055 | 0.219 |

**Table S2.** Comparison of bacterial community richness (observed features [ASVs]) in the cecal luminal microbiota of birds from 4 genetic lines and 2 conditions (thermoneutral [TN] or heat stressed [HS]) using the Kruskal-Wallis test.

| **Kruskal-Wallis** | **H** | **p-value** |
| --- | --- | --- |
| All groups | 30.9 | <0.001 |

| **Group 1** | **Group 2** | **H** | **p-value** | **q-value** |
| --- | --- | --- | --- | --- |
| L1995-HS (n=6) | L1995-TN (n=6) | 8.31 | 0.004 | 0.023 |
|  | L2015-HS (n=5) | 5.63 | 0.018 | 0.033 |
|  | L2015-TN (n=6) | 8.31 | 0.004 | 0.023 |
|  | ACRB-HS (n=6) | 6.56 | 0.010 | 0.025 |
|  | ACRB-TN (n=5) | 1.20 | 0.273 | 0.364 |
|  | JF-HS (n=6) | 7.41 | 0.006 | 0.023 |
|  | JF-TN (n=6) | 0.64 | 0.423 | 0.474 |
| L1995-TN (n=6) | L2015-HS (n=5) | 7.50 | 0.006 | 0.023 |
|  | L2015-TN (n=6) | 0.64 | 0.423 | 0.474 |
|  | ACRB-HS (n=6) | 0.23 | 0.631 | 0.654 |
|  | ACRB-TN (n=5) | 6.53 | 0.011 | 0.025 |
|  | JF-HS (n=6) | 1.26 | 0.262 | 0.364 |
|  | JF-TN (n=6) | 6.56 | 0.010 | 0.025 |
| L2015-HS (n=5) | L2015-TN (n=6) | 7.50 | 0.006 | 0.023 |
|  | ACRB-HS (n=6) | 7.50 | 0.006 | 0.023 |
|  | ACRB-TN (n=5) | 0.88 | 0.347 | 0.423 |
|  | JF-HS (n=6) | 7.50 | 0.006 | 0.023 |
|  | JF-TN (n=6) | 2.13 | 0.144 | 0.224 |
| L2015-TN (n=6) | ACRB-HS (n=6) | 0.23 | 0.631 | 0.654 |
|  | ACRB-TN (n=5) | 5.63 | 0.018 | 0.033 |
|  | JF-HS (n=6) | 1.64 | 0.200 | 0.295 |
|  | JF-TN (n=6) | 4.33 | 0.037 | 0.062 |
| ACRB-HS (n=6) | ACRB-TN (n=5) | 4.80 | 0.028 | 0.050 |
|  | JF-HS (n=6) | 0.92 | 0.337 | 0.423 |
|  | JF-TN (n=6) | 5.77 | 0.016 | 0.033 |
| ACRB-TN (n=5) | JF-HS (n=6) | 6.53 | 0.011 | 0.025 |
|  | JF-TN (n=6) | 0.03 | 0.855 | 0.855 |
| JF-HS (n=6) | JF-TN (n=6) | 7.41 | 0.006 | 0.023 |

**Table S3.** Comparison of bacterial community richness (Faith’s phylogenetic diversity) in the cecal luminal microbiota of birds from 4 genetic lines and 2 conditions (thermoneutral [TN] or heat stressed [HS]) using the Kruskal-Wallis test.

| **Kruskal-Wallis** | **H** | **p-value** |
| --- | --- | --- |
| All groups | 26.0 | <0.001 |

| **Group 1** | **Group 2** | **H** | **p-value** | **q-value** |
| --- | --- | --- | --- | --- |
| L1995-HS (n=6) | L1995-TN (n=6) | 6.56 | 0.010 | 0.042 |
|  | L2015-HS (n=5) | 2.70 | 0.100 | 0.156 |
|  | L2015-TN (n=6) | 7.41 | 0.006 | 0.036 |
|  | ACRB-HS (n=6) | 7.41 | 0.006 | 0.036 |
|  | ACRB-TN (n=5) | 0.03 | 0.855 | 0.940 |
|  | JF-HS (n=6) | 5.77 | 0.016 | 0.045 |
|  | JF-TN (n=6) | 0.10 | 0.749 | 0.874 |
| L1995-TN (n=6) | L2015-HS (n=5) | 7.50 | 0.006 | 0.036 |
|  | L2015-TN (n=6) | 0.00 | 1.000 | 1.000 |
|  | ACRB-HS (n=6) | 0.10 | 0.749 | 0.874 |
|  | ACRB-TN (n=5) | 5.63 | 0.018 | 0.045 |
|  | JF-HS (n=6) | 0.00 | 1.000 | 1.000 |
|  | JF-TN (n=6) | 4.33 | 0.037 | 0.075 |
| L2015-HS (n=5) | L2015-TN (n=6) | 7.50 | 0.006 | 0.036 |
|  | ACRB-HS (n=6) | 7.50 | 0.006 | 0.036 |
|  | ACRB-TN (n=5) | 3.15 | 0.076 | 0.129 |
|  | JF-HS (n=6) | 6.53 | 0.011 | 0.042 |
|  | JF-TN (n=6) | 2.13 | 0.144 | 0.212 |
| L2015-TN (n=6) | ACRB-HS (n=6) | 0.41 | 0.522 | 0.731 |
|  | ACRB-TN (n=5) | 5.63 | 0.018 | 0.045 |
|  | JF-HS (n=6) | 0.23 | 0.631 | 0.803 |
|  | JF-TN (n=6) | 3.69 | 0.055 | 0.102 |
| ACRB-HS (n=6) | ACRB-TN (n=5) | 5.63 | 0.018 | 0.045 |
|  | JF-HS (n=6) | 0.03 | 0.873 | 0.940 |
|  | JF-TN (n=6) | 4.33 | 0.037 | 0.075 |
| ACRB-TN (n=5) | JF-HS (n=6) | 4.80 | 0.028 | 0.066 |
|  | JF-TN (n=6) | 0.30 | 0.584 | 0.779 |
| JF-HS (n=6) | JF-TN (n=6) | 3.10 | 0.078 | 0.129 |

**Table S4.** Comparison of bacterial community evenness in the cecal luminal microbiota of birds from 4 genetic lines and 2 conditions (thermoneutral [TN] or heat stressed [HS]) using the Kruskal-Wallis test.

| **Kruskal-Wallis** | **H** | **p-value** |
| --- | --- | --- |
| All groups | 15.0 | 0.035 |

| **Group 1** | **Group 2** | **H** | **p-value** | **q-value** |
| --- | --- | --- | --- | --- |
| L1995-HS (n=6) | L1995-TN (n=6) | 0.03 | 0.873 | 0.905 |
|  | L2015-HS (n=5) | 1.20 | 0.273 | 0.547 |
|  | L2015-TN (n=6) | 0.41 | 0.522 | 0.664 |
|  | ACRB-HS (n=6) | 0.03 | 0.873 | 0.905 |
|  | ACRB-TN (n=5) | 0.13 | 0.715 | 0.870 |
|  | JF-HS (n=6) | 0.92 | 0.337 | 0.589 |
|  | JF-TN (n=6) | 1.64 | 0.200 | 0.431 |
| L1995-TN (n=6) | L2015-HS (n=5) | 6.53 | 0.011 | 0.074 |
|  | L2015-TN (n=6) | 1.64 | 0.200 | 0.431 |
|  | ACRB-HS (n=6) | 0.00 | 1.000 | 1.000 |
|  | ACRB-TN (n=5) | 0.03 | 0.855 | 0.905 |
|  | JF-HS (n=6) | 0.64 | 0.423 | 0.593 |
|  | JF-TN (n=6) | 3.10 | 0.078 | 0.274 |
| L2015-HS (n=5) | L2015-TN (n=6) | 3.33 | 0.068 | 0.272 |
|  | ACRB-HS (n=6) | 2.70 | 0.100 | 0.312 |
|  | ACRB-TN (n=5) | 5.77 | 0.016 | 0.091 |
|  | JF-HS (n=6) | 6.53 | 0.011 | 0.074 |
|  | JF-TN (n=6) | 7.50 | 0.006 | 0.074 |
| L2015-TN (n=6) | ACRB-HS (n=6) | 0.64 | 0.423 | 0.593 |
|  | ACRB-TN (n=5) | 2.13 | 0.144 | 0.367 |
|  | JF-HS (n=6) | 4.33 | 0.037 | 0.174 |
|  | JF-TN (n=6) | 6.56 | 0.010 | 0.074 |
| ACRB-HS (n=6) | ACRB-TN (n=5) | 0.03 | 0.855 | 0.905 |
|  | JF-HS (n=6) | 0.92 | 0.337 | 0.589 |
|  | JF-TN (n=6) | 0.64 | 0.423 | 0.593 |
| ACRB-TN (n=5) | JF-HS (n=6) | 0.83 | 0.361 | 0.593 |
|  | JF-TN (n=6) | 2.13 | 0.144 | 0.367 |
| JF-HS (n=6) | JF-TN (n=6) | 0.41 | 0.522 | 0.664 |

**Table S5.** Comparisons of all groups in the cecal mucosal microbiota of birds using the Kruskal-Wallis test with 4 alpha diversity metrics. As groups were not considered significantly different overall, pairwise comparisons were not utilized.

| **Alpha diversity metric** | **H** | **p-value** |
| --- | --- | --- |
| Shannon diversity | 7.44 | 0.385 |
| Observed features (ASVs) | 8.77 | 0.270 |
| Faith’s phylogenetic diversity | 9.88 | 0.195 |
| Evenness | 9.98 | 0.190 |

**Table S6.** Group and pairwise PERMANOVA statistics based on the unweighted UniFrac distance matrix on cecal luminal microbiota samples.

| **PERMANOVA** | **# of permutations** | **pseudo-F** | **p-value** |
| --- | --- | --- | --- |
| All groups | 999 | 2.11 | 0.001 |

| **Group 1** | **Group 2** | **pseudo-F** | **p-value** | **q-value** |
| --- | --- | --- | --- | --- |
| L1995-HS (n=5) | L1995-TN (n=4) | 2.37 | 0.003 | 0.012 |
|  | L2015-HS (n=5) | 1.82 | 0.009 | 0.018 |
|  | L2015-TN (n=5) | 2.19 | 0.002 | 0.012 |
|  | ACRB-HS (n=6) | 3.13 | 0.002 | 0.012 |
|  | ACRB-TN (n=5) | 2.08 | 0.003 | 0.012 |
|  | JF-HS (n=6) | 3.14 | 0.004 | 0.012 |
|  | JF-TN (n=4) | 1.66 | 0.007 | 0.016 |
| L1995-TN (n=4) | L2015-HS (n=5) | 2.95 | 0.004 | 0.012 |
|  | L2015-TN (n=5) | 0.98 | 0.557 | 0.557 |
|  | ACRB-HS (n=6) | 1.33 | 0.198 | 0.231 |
|  | ACRB-TN (n=5) | 2.63 | 0.001 | 0.012 |
|  | JF-HS (n=6) | 1.89 | 0.009 | 0.018 |
|  | JF-TN (n=4) | 2.07 | 0.014 | 0.022 |
| L2015-HS (n=5) | L2015-TN (n=5) | 2.71 | 0.006 | 0.015 |
|  | ACRB-HS (n=6) | 2.88 | 0.005 | 0.014 |
|  | ACRB-TN (n=5) | 2.09 | 0.012 | 0.020 |
|  | JF-HS (n=6) | 3.75 | 0.003 | 0.012 |
|  | JF-TN (n=4) | 2.03 | 0.011 | 0.019 |
| L2015-TN (n=5) | ACRB-HS (n=6) | 1.13 | 0.260 | 0.280 |
|  | ACRB-TN (n=5) | 2.61 | 0.003 | 0.012 |
|  | JF-HS (n=6) | 1.68 | 0.019 | 0.027 |
|  | JF-TN (n=4) | 1.89 | 0.020 | 0.027 |
| ACRB-HS (n=6) | ACRB-TN (n=5) | 2.13 | 0.016 | 0.024 |
|  | JF-HS (n=6) | 1.19 | 0.246 | 0.276 |
|  | JF-TN (n=4) | 1.59 | 0.087 | 0.106 |
| ACRB-TN (n=5) | JF-HS (n=6) | 2.57 | 0.011 | 0.019 |
|  | JF-TN (n=4) | 1.10 | 0.283 | 0.293 |
| JF-HS (n=6) | JF-TN (n=4) | 2.03 | 0.040 | 0.051 |

**Table S7.** Group and pairwise PERMANOVA statistics based on the weighted UniFrac distance matrix on cecal luminal microbiota samples.

| **PERMANOVA** | **# of permutations** | **pseudo-F** | **p-value** |
| --- | --- | --- | --- |
| All groups | 999 | 1.59 | 0.011 |

| **Group 1** | **Group 2** | **pseudo-F** | **p-value** | **q-value** |
| --- | --- | --- | --- | --- |
| L1995-HS (n=5) | L1995-TN (n=4) | 0.40 | 0.919 | 0.919 |
|  | L2015-HS (n=5) | 1.18 | 0.288 | 0.448 |
|  | L2015-TN (n=5) | 1.47 | 0.171 | 0.342 |
|  | ACRB-HS (n=6) | 1.34 | 0.214 | 0.375 |
|  | ACRB-TN (n=5) | 0.73 | 0.539 | 0.580 |
|  | JF-HS (n=6) | 1.28 | 0.270 | 0.445 |
|  | JF-TN (n=4) | 1.95 | 0.106 | 0.261 |
| L1995-TN (n=4) | L2015-HS (n=5) | 0.97 | 0.475 | 0.532 |
|  | L2015-TN (n=5) | 1.85 | 0.086 | 0.261 |
|  | ACRB-HS (n=6) | 1.17 | 0.323 | 0.452 |
|  | ACRB-TN (n=5) | 1.00 | 0.368 | 0.477 |
|  | JF-HS (n=6) | 1.60 | 0.112 | 0.261 |
|  | JF-TN (n=4) | 2.64 | 0.003 | 0.042 |
| L2015-HS (n=5) | L2015-TN (n=5) | 2.08 | 0.055 | 0.248 |
|  | ACRB-HS (n=6) | 0.98 | 0.390 | 0.477 |
|  | ACRB-TN (n=5) | 1.05 | 0.425 | 0.496 |
|  | JF-HS (n=6) | 1.66 | 0.144 | 0.310 |
|  | JF-TN (n=4) | 1.85 | 0.079 | 0.261 |
| L2015-TN (n=5) | ACRB-HS (n=6) | 1.80 | 0.098 | 0.261 |
|  | ACRB-TN (n=5) | 2.25 | 0.025 | 0.140 |
|  | JF-HS (n=6) | 2.99 | 0.006 | 0.056 |
|  | JF-TN (n=4) | 5.21 | 0.002 | 0.042 |
| ACRB-HS (n=6) | ACRB-TN (n=5) | 1.04 | 0.392 | 0.477 |
|  | JF-HS (n=6) | 1.12 | 0.306 | 0.451 |
|  | JF-TN (n=4) | 2.17 | 0.062 | 0.248 |
| ACRB-TN (n=5) | JF-HS (n=6) | 1.46 | 0.208 | 0.375 |
|  | JF-TN (n=4) | 0.61 | 0.802 | 0.832 |
| JF-HS (n=6) | JF-TN (n=4) | 2.83 | 0.017 | 0.119 |

**Table S8.** Group and pairwise PERMANOVA statistics based on the unweighted UniFrac distance matrix on cecal mucosal microbiota samples.

| **PERMANOVA** | **# of permutations** | **pseudo-F** | **p-value** |
| --- | --- | --- | --- |
| All groups | 999 | 1.40 | 0.005 |

| **Group 1** | **Group 2** | **pseudo-F** | **p-value** | **q-value** |
| --- | --- | --- | --- | --- |
| L1995-HS (n=5) | L1995-TN (n=4) | 1.13 | 0.294 | 0.392 |
|  | L2015-HS (n=5) | 2.49 | 0.012 | 0.112 |
|  | L2015-TN (n=5) | 1.53 | 0.026 | 0.152 |
|  | ACRB-HS (n=6) | 1.81 | 0.011 | 0.112 |
|  | ACRB-TN (n=5) | 2.32 | 0.011 | 0.112 |
|  | JF-HS (n=6) | 1.77 | 0.078 | 0.168 |
|  | JF-TN (n=4) | 1.92 | 0.028 | 0.152 |
| L1995-TN (n=4) | L2015-HS (n=5) | 1.80 | 0.037 | 0.152 |
|  | L2015-TN (n=5) | 1.09 | 0.322 | 0.410 |
|  | ACRB-HS (n=6) | 1.38 | 0.110 | 0.205 |
|  | ACRB-TN (n=5) | 1.69 | 0.044 | 0.154 |
|  | JF-HS (n=6) | 1.83 | 0.072 | 0.168 |
|  | JF-TN (n=4) | 1.31 | 0.183 | 0.301 |
| L2015-HS (n=5) | L2015-TN (n=5) | 1.61 | 0.038 | 0.152 |
|  | ACRB-HS (n=6) | 1.09 | 0.291 | 0.392 |
|  | ACRB-TN (n=5) | 1.37 | 0.094 | 0.188 |
|  | JF-HS (n=6) | 1.59 | 0.068 | 0.168 |
|  | JF-TN (n=4) | 0.92 | 0.558 | 0.601 |
| L2015-TN (n=5) | ACRB-HS (n=6) | 1.21 | 0.194 | 0.302 |
|  | ACRB-TN (n=5) | 1.46 | 0.074 | 0.168 |
|  | JF-HS (n=6) | 1.67 | 0.056 | 0.168 |
|  | JF-TN (n=4) | 1.29 | 0.174 | 0.301 |
| ACRB-HS (n=6) | ACRB-TN (n=5) | 1.03 | 0.405 | 0.493 |
|  | JF-HS (n=6) | 0.94 | 0.494 | 0.553 |
|  | JF-TN (n=4) | 0.87 | 0.591 | 0.613 |
| ACRB-TN (n=5) | JF-HS (n=6) | 0.94 | 0.461 | 0.538 |
|  | JF-TN (n=4) | 0.89 | 0.635 | 0.635 |
| JF-HS (n=6) | JF-TN (n=4) | 1.18 | 0.228 | 0.336 |

**Table S9.** Group and pairwise PERMANOVA statistics based on the weighted UniFrac distance matrix on cecal mucosal microbiota samples.

| **PERMANOVA** | **# of permutations** | **pseudo-F** | **p-value** |
| --- | --- | --- | --- |
| All groups | 999 | 1.46 | 0.031 |

| **Group 1** | **Group 2** | **pseudo-F** | **p-value** | **q-value** |
| --- | --- | --- | --- | --- |
| L1995-HS (n=5) | L1995-TN (n=4) | 0.91 | 0.473 | 0.530 |
|  | L2015-HS (n=5) | 0.95 | 0.418 | 0.488 |
|  | L2015-TN (n=5) | 1.11 | 0.341 | 0.450 |
|  | ACRB-HS (n=6) | 0.86 | 0.557 | 0.600 |
|  | ACRB-TN (n=5) | 1.21 | 0.254 | 0.450 |
|  | JF-HS (n=6) | 0.78 | 0.660 | 0.660 |
|  | JF-TN (n=4) | 1.25 | 0.292 | 0.450 |
| L1995-TN (n=4) | L2015-HS (n=5) | 1.62 | 0.118 | 0.403 |
|  | L2015-TN (n=5) | 1.25 | 0.318 | 0.450 |
|  | ACRB-HS (n=6) | 1.16 | 0.322 | 0.450 |
|  | ACRB-TN (n=5) | 0.82 | 0.634 | 0.657 |
|  | JF-HS (n=6) | 1.99 | 0.010 | 0.154 |
|  | JF-TN (n=4) | 1.99 | 0.133 | 0.403 |
| L2015-HS (n=5) | L2015-TN (n=5) | 1.54 | 0.167 | 0.403 |
|  | ACRB-HS (n=6) | 1.25 | 0.266 | 0.450 |
|  | ACRB-TN (n=5) | 2.96 | 0.024 | 0.224 |
|  | JF-HS (n=6) | 1.25 | 0.280 | 0.450 |
|  | JF-TN (n=4) | 1.02 | 0.370 | 0.450 |
| L2015-TN (n=5) | ACRB-HS (n=6) | 1.40 | 0.164 | 0.403 |
|  | ACRB-TN (n=5) | 1.69 | 0.071 | 0.369 |
|  | JF-HS (n=6) | 2.33 | 0.011 | 0.154 |
|  | JF-TN (n=4) | 1.53 | 0.187 | 0.403 |
| ACRB-HS (n=6) | ACRB-TN (n=5) | 1.62 | 0.147 | 0.403 |
|  | JF-HS (n=6) | 1.05 | 0.370 | 0.450 |
|  | JF-TN (n=4) | 1.27 | 0.323 | 0.450 |
| ACRB-TN (n=5) | JF-HS (n=6) | 2.40 | 0.051 | 0.357 |
|  | JF-TN (n=4) | 2.06 | 0.079 | 0.369 |
| JF-HS (n=6) | JF-TN (n=4) | 1.60 | 0.177 | 0.403 |
